# Environmental Enrichment Reverses Maternal Sleep Deprivation-Induced Anxiety-Like Behavior and Cognitive Impairment in CD-1 Mice

**DOI:** 10.3389/fnbeh.2022.943900

**Published:** 2022-07-13

**Authors:** Yue-Ming Zhang, Yun-Zhou Cheng, Ya-Tao Wang, Ru-Meng Wei, Yi-Jun Ge, Xiao-Yi Kong, Xue-Yan Li

**Affiliations:** ^1^Department of Neurology (Sleep Disorders), The Affiliated Chaohu Hospital of Anhui Medical University, Hefei, China; ^2^Department of Pediatrics, The Affiliated Chaohu Hospital of Anhui Medical University, Hefei, China

**Keywords:** maternal sleep deprivation, learning and memory, BDNF, synaptotagmin-1, enriched environment

## Abstract

Preclinical studies have clearly indicated that offspring of mothers who suffered sleep deprivation during pregnancy exhibit anxiety, depression-like behaviors, and cognitive deficits. The cognitive impairment induced by maternal sleep deprivation (MSD) is currently poorly treated. Growing evidence indicates that an enriched environment (EE) improves cognition function in models of Alzheimer’s disease, schizophrenia, and lipopolysaccharide. However, the effects of EE on hippocampal-dependent learning and memory, as well as synaptic plasticity markers changes induced by MSD, are unclear. In the present study, pregnant CD-1 mice were randomly divided into a control group, MSD group, and MSD+EE group. Two different living environments, including standard environment and EE, were prepared. When male and female offspring were 2 months, the open field test and elevated plus maze were used to assess anxiety-like behavior, and the Morris water maze was used to evaluate hippocampal learning and memory. Western blotting and real-time fluorescence quantitative polymerase chain reaction were used to detect the expression of brain-derived neurotrophic factor and Synaptotagmin-1 in the hippocampus of offspring. The results revealed that MSD-induced offspring showed anxiety-like behaviors and cognitive impairment, while EE alleviated anxiety-like behavior and cognitive impairment in offspring of the MSD+EE group. The cognitive impairment induced by MSD was associated with a decreased brain-derived neurotrophic factor and an increased Synaptotagmin-1, while EE increased and decreased brain-derived neurotrophic factor and Synaptotagmin-1 in the hippocampus of mice from the MSD+EE group, respectively. Taken together, we can conclude that EE has beneficial effects on MSD-induced synaptic plasticity markers changes and can alleviate anxiety-like behaviors and cognitive impairment.

## Introduction

Sleep is a conservative behavior of mammals. Adequate sleep is conducive to energy recovery, removal of toxic substances, and consolidation of learning and memory (Xie et al., 2013). However, in modern society, lack of sleep has become a prevalent phenomenon in many populations, especially for women during pregnancy (Sedov et al., 2018). Epidemiological studies have shown that about half of pregnant women complained about their sleep, which was characterized by decreased total sleep time, sleep efficiency, rapid eye movement sleep, and slow-wave sleep (Wilson et al., 2011). Notably, the abnormal sleep pattern in the third trimester of pregnancy has been ascribed to conditions such as nocturia, nausea, discomfort from fetal movements, difficulty in assuming usual sleep positions, back pain, and hormonal oscillations, and can lead to chronic sleep deprivation (Izci-Balserak et al., 2018). Concurrently, the third trimester of pregnancy is also a critical time for fetal brain and center nervous system development (Micheli et al., 2011). Clinical studies have found that chronic sleep deprivation not only increases the risk of psychiatric disorders in mothers but also has a series of adverse effects on the offspring (Chang et al., 2010). Due to ethical limitations, the underlying mechanisms can be explored through animal models of sleep restriction during pregnancy.

Similarly to many animal models of early life stress, the maternal sleep deprivation (MSD) model affects fetal hippocampal synapse development by disrupting the intrauterine environment (Entringer et al., 2012). Previous studies have found that MSD-induced rats showed hippocampal-dependent memory impairment, which was associated with decreased hippocampal neurogenesis and increased levels of pro-inflammatory markers due to microglial activation (Zhao et al., 2014). It is known that hippocampal synaptic plasticity indicated with long-term potentiation (LTP) is a cellular mechanism underlying information processing and memory formation (Yang et al., 2021). The offspring of mothers subjected to sleep deprivation at different stages of pregnancy have been found to exhibit anxiety, depressive-like behaviors, and cognitive deficits, accompanied by impaired LTP and basal vesicle transmission in the CA1 region of the hippocampus (Peng et al., 2016). Brain-derived neurotrophic factor (BDNF) and Synaptotagmin-1 (Syt-1) are two important synaptic plasticity markers, which have been experimentally confirmed to be associated with cognitive impairment induced by early life stress (Thome et al., 2001; Leal et al., 2015). BDNF, a main neurotrophin in mammals’ hippocampus, is essential for synaptic transmission and regulates dendritic arborization and LTP, while Syt-1, a Ca^2+^-dependent synaptic protein, can bind with SNARE (soluble N-ethylmaleimide-sensitive factor attachment protein receptor) complex and directly interacts with SNAP-25 (synaptosomal-associated protein of 25 kDa) on a presynaptic membrane to facilitate neurotransmitter release (Spriggs et al., 2019; Chanaday et al., 2021). Collectively, gestational sleep deprivation causes emotional and cognitive dysfunction in offspring, but whether the mechanisms of cognitive dysfunction involve changes in synaptic plasticity markers is unclear.

The concept of an enriched environment (EE) was proposed by Hebb in 1947 and is a simple and effective method to improve cognitive deficits (Alwis and Rajan, 2014). EE is an experimental paradigm that allows mice to receive sensory, motor, and social stimulation by placing them in larger devices equipped with a variety of toys and running wheels (Yu et al., 2020; Zhang et al., 2021). EE has been found to affect cell survival, neurogenesis, synaptogenesis, and dendritic morphology in the hippocampus (Wang et al., 2020). Several findings have indicated that EE improves activity-dependent synaptic plasticity, LTP, social interaction, and spatial learning and memory in models of Alzheimer’s disease, brain injury, Parkinson’s disease, and schizophrenia (Murueta-Goyena et al., 2019). Our lab has also shown that long-term EE attenuates the exacerbated age-related cognitive impairment induced by lipopolysaccharide exposure during pregnancy (Zhuang et al., 2021).

In this study, we investigated the effects of EE on anxiety and spatial learning and memory in MSD-induced male and female offspring *via* increased dwelling space containing novel toys at the end of lactation. This study provides evidence for the establishment of EE as an additional therapy option for improving brain functioning in offspring who have suffered from MSD.

## Methods

### Animals

Both male and female CD-1 mice (8 weeks) were purchased from Beijing Vital River Laboratory Animal Device Co., Ltd. (SPF grade). The animals were housed in individual cages maintained at a temperature of 22–25°C, a 12 h dark-light photoperiod, and 60%–70% relative humidity. Food and water were available ad libitum. All procedures were carried out in compliance with the guidelines for humane treatment set by the Association of Laboratory Animal Sciences and the Center for Laboratory Animal Sciences at Anhui Medical University.

### Experimental Protocols

**Figure 1:**
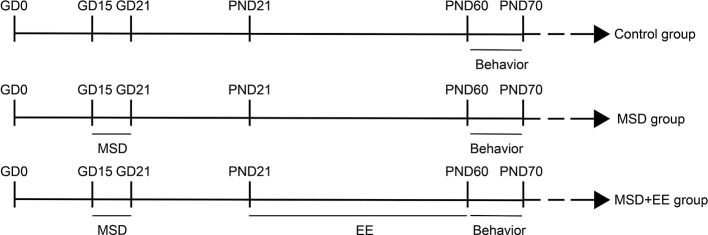
Timeline of the experiment (see “Experimental Protocols” Section for details).

The male and female CD-1 mice were acclimated to the new environment for 2 weeks, and were then paired at a ratio of 1:2. The 30 female mice with vaginal smear were selected and randomly assigned to one of the three following groups: (1) control group (*n* = 10), (2) MSD group (*n* = 10), and (3) MSD+EE group (*n* = 10). Pregnant mice of the MSD and MSD+EE groups were put into the BW-NSD404 sleep deprivation machine (Shanghai Bio-will Co., Ltd.) during the third period (GD15-GD21) of pregnancy from 12:00 to 18:00. The sleep deprivation machine ensured that mice remained awake during deprivation by the continuous work of the running belt and the speed of the running belt was set to 0.5 m/min. Concurrently, the pregnant mice of the Control group were put into the other BW-NSD404 sleep deprivation machine at a speed of 0 m/min. The mice from the three groups lived in the same environment during and after sleep deprivation. Mice had free access to food and water throughout the sleep deprivation period. After weaning, the offspring from the MSD+EE group (*n* = 15) were raised in larger cages (52 × 40 × 20 cm) with 7–8 mice/cage containing various colorful toys, including platforms, a wood shelter, running wheels, ladders, and plastic tunnels. Objects were changed twice a week. The offspring from the MSD group (*n* = 15) and the Control group (*n* = 15) were raised in standard cages (36 × 18 × 14 cm) with three mice/cage without objects. At 2 months of age, all offspring were examined by behavioral and molecular experiments (Figure 1).

### Open Field Test

In the open field test (OFT), mice were gently placed in the center of a black wooden box (50 cm × 50 cm × 25 cm) for 5 min, and exploratory behavior was automatically recorded by the ANY-maze video tracking system (Stoeling, USA). Time spent in and the number of entries into the central area and total distance were recorded by ANY-maze software. After each test, the arena was cleaned with 75% alcohol to avoid the interference of odor.

### Elevated Plus Maze

The elevated plus maze (EPM) consisted of two open arms and two closed arms arranged at right angles. The height of the maze was about 50 cm above the ground. At the beginning of the experiment, mice were put into the central area of the maze with their head facing the open arms. The number of entries and the time spent in each arm were recorded for 5 min by the ANY-maze video tracking system. After the recording, the maze was wiped with 75% alcohol to eliminate the odor of the mouse.

### Morris Water Maze

The Morris water maze (MWM) was used to assess the spatial learning and memory abilities of mice. The protocol used in this study was similar to those described previously (Wu et al., 2020). The test was divided into two parts: a learning phase and a memory phase. During the learning phase, mice were trained in a circular black pool (diameter 120 cm, height 30 cm) over four trials per day for seven consecutive days to find the hidden platform. Mice were allowed to rest on the platform for 30 s if they failed to find the hidden platform within 60 s. During the memory phase, a probe trial task was performed after the hidden platform had been removed. All trials were recorded and analyzed using the ANY-maze tracking system.

### Western Blotting

The Western blotting procedure was performed as previously described (Zhuang et al., 2021). Hippocampal tissue was lysed in RIRA lysis buffer, and protein concentrations were determined using a BCA Protein Assay Kit. The protein samples were electrophoretically separated and then blotted onto nitrocellulose membranes. Protein levels were determined *via* incubation against antibodies of BDNF (1:1,000; Abcam, Cambridge, UK) and Syt-1 (1:1,000; Bioss, Beijing, China). Bands were visualized by enhanced chemiluminescence and quantified using ImageJ software.

### Real-Time Fluorescence Quantitative Polymerase Chain Reaction

Total RNA was extracted from the hippocampal tissue by adding Trizol lysate. The purity of the extracted RNA was assessed using a spectrophotometer. RNA was reverse-transcribed to cDNA using the ReverAidTM First-Strand cDNA Synthesis Kit. The transcripts were amplified by quantitative real-time polymerase chain reaction. The reaction system consisted of 5 μl of 2× SYBR Green Mixture, 1 μl of upstream primer, 1 μl of downstream primer, 1 μl of cDNA, and 2 μl of RNase Free water. The reaction conditions were as follows: a single cycle of pre-denaturation at 95°C for 1 min, and a total of 40 cycles at 95°C for 20 s and 60°C for 1 min. The primer sequence is shown in Table 1.

**Table 1:** Primer sequences.

Gene	Forward primer (5′3′)	Reverse primer (5′3′)
β-actin	AGTGTGACGTTGACATCCGT	TGCTAGGAGCCAGAGCAGTA
BDNF	TTACTCTCCTGGGTTCCTGA	ACGTCCACTTCTGTTTCCTT
Syt-1	GTCCTTCTAGTCGTGACCTG	GCCTGATCCTTCATGGTCTT

### Data Analysis

All values are expressed as the mean ± standard error of the mean. Repeated-measure analysis of variance (ANOVA) was used to analyze data from the MWM test. Data of anxiety-like behaviors, cognitive cognition, and synaptic plasticity markers were analyzed using a two-way analysis of variance with Tukey’s least-significant difference *post-hoc* test to compare differences between the three groups. Differences were considered significant at *P* < 0.05. All data analyses were performed using GraphPad 8.0.

## Results

### Environmental Enrichment Reverses Anxiety-Like Behavior Induced by Maternal Sleep Deprivation

**Figure 2:**
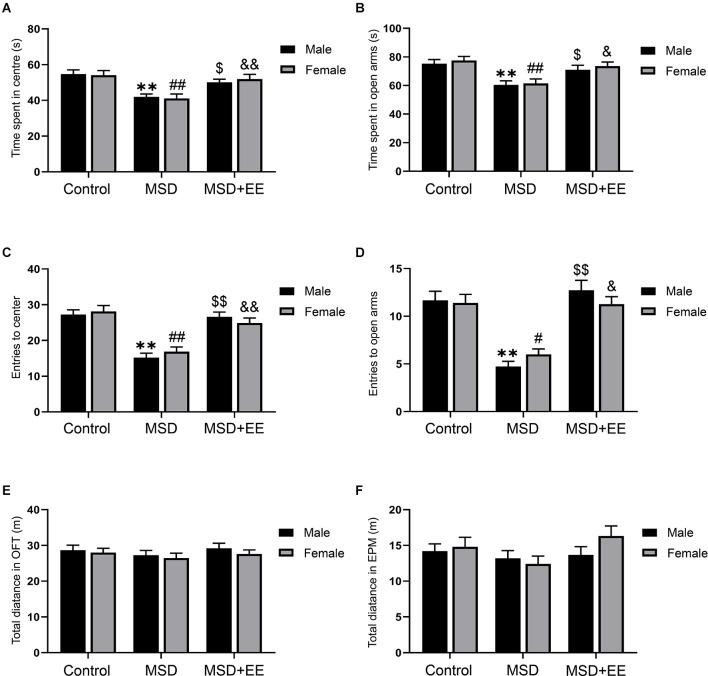
The effect of EE and MSD on anxiety-like behaviors. **(A)** The time spent in the center of the three groups during the open field test. **(B)** The time spent in open arms of the three groups during the elevated plus maze. **(C)** The number of the entries to the center of the open field test. **(D)** The number of the entries to the open arms of the elevated plus maze. **(E)** The total distance of the open field test. **(F)** The total distance of the elevated plus maze. **P < 0.01 vs. control male; ^#^*P* < 0.05, ^##^*P* < 0.01 vs. control female; ^$^*P* < 0.05, ^$$^*P* < 0.01 vs. MSD male; ^&^*P* < 0.05, ^&&^*P* < 0.01 vs. MSD female.

First, we evaluated the effect of MSD on anxiety-like behaviors using the OFT and EPM, and assess the potential therapeutic effects of EE. Two-way ANOVA revealed a significant treatment effect for center time (*F*_(2,84)_ = 16.97, *P* < 0.01; Figure 2A) and number of the center entries (*F*_(2,84)_ = 40.42, *P* < 0.01; Figure 2C) among the three groups during the OFT. The *post hoc* analysis confirmed that the mice from the MSD group exhibited more anxiety-like behaviors than the control group (*P*s < 0.05), and EE reversed this abnormal mental state (*P*s < 0.05). Similarly, a two-way ANOVA revealed significant between-group differences in open arms time (*F*_(2,84)_ = 14.69, *P* < 0.01; Figure 2B) and number of the open arms entries (*F*_(2,84)_ = 41.23, *P* < 0.01; Figure 2D) during the EPM. * Post hoc* analysis revealed that EE normalized the anxiety-like behavior associated with MSD (*P*s < 0.05). There were no differences in the total distance among the three groups during the OFT and EPM, which indicated that MSD did not impair the motor ability of offspring (Figures 2E,F). Collectively, the OFT and EPM results indicated that EE had a healing effect on anxiety-like behaviors caused by MSD.

### Environmental Enrichment Improves Cognitive Impairment Induced by Maternal Sleep Deprivation

**Figure 3:**
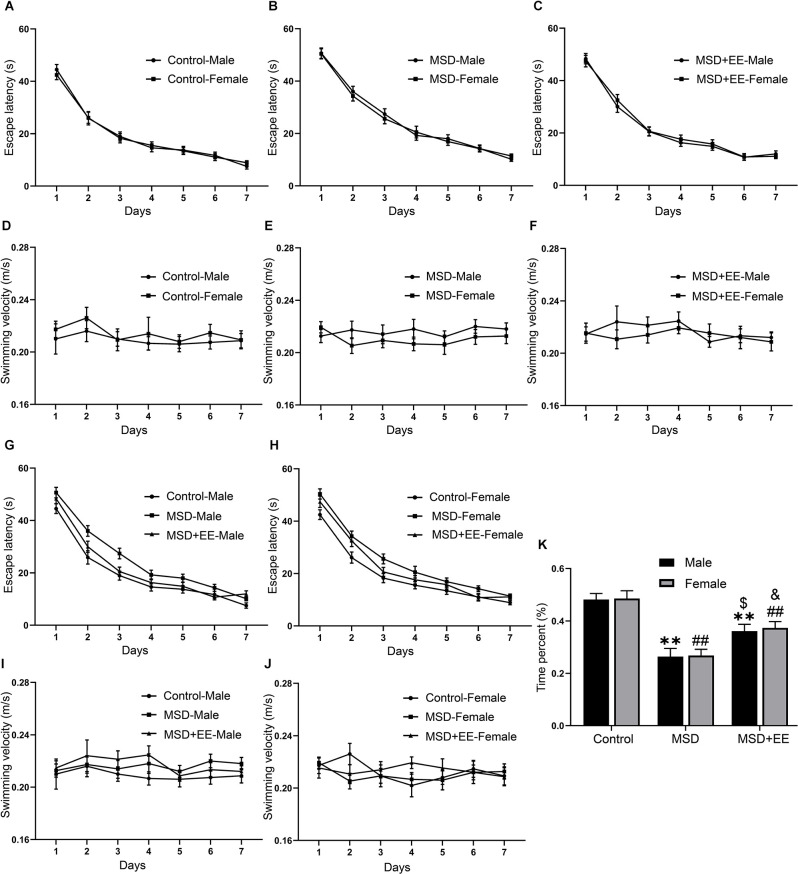
EE attenuated the decline in spatial learning and memory ability of MSD-induced mice. In the learning phase, the escape latency **(A,B,C,G,H)** and swimming velocity **(D,E,F,I,J)** for each of the seven training days are shown. In the memory phase, the percent time spent in the target quadrant is shown **(K)**. **P < 0.01 vs. control male; ^##^*P* < 0.01 vs. control female; ^$^*P* < 0.05 vs. MSD male; ^&^*P* < 0.05 vs. MSD female.

The MWM was used to evaluate the effect of EE on MSD-induced cognitive deficits. In the learning phase, a repeated-measures ANOVA revealed no sex differences in escape latency (Control group: *F*_(1,28)_ = 0.05, *P* > 0.05; Figure 3A; MSD group: *F*_(1,28)_ = 0.14, *P* > 0.05; Figure 3B; MSD+EE group: *F*_(1,28)_ = 0.24, *P* > 0.05; Figure 3C) and swimming velocity (Control group: *F*_(1,28)_ = 0.98, *P* > 0.05; Figure 3D; MSD group: *F*_(1,28)_ = 3.08, *P* > 0.05; Figure 3E; MSD+EE group: *F*_(1,28)_ = 1.00, *P* > 0.05; Figure 3F) for each group when the analysis was controlled for treatment. However, a repeated-measures ANOVA for latency from different groups showed significant differences when the analysis was controlled for sex (male group: *F*_(2,42)_ = 23.19, *P* < 0.01; Figure 3G; female: *F*_(2,42)_ = 16.39, *P* < 0.01; Figure 3H). The *post hoc* analysis revealed that both male and female mice from the MSD group spent more time to find the hidden platform than those in the control group (*P*s < 0.01), while both male and female mice from the MSD+EE group spent less time to find the hidden platform than those in the MSD group (*P*s < 0.05). There were no sex differences in swimming velocity within any of the three groups (male group: *F*_(2,42)_ = 3.24, *P* = 0.05; Figure 3I; female group: *F*_(2,42)_ = 0.38, *P* > 0.05; Figure 3J).

In the memory phase, the two-way ANOVA showed that the time spent in the target quadrant was significantly different between the three groups (*F*_(2,84)_ = 34.30, *P* < 0.01; Figure 3K). *Post hoc* comparisons showed the time percent was lowest in the MSD group (*Ps* < 0.05). EE could ameliorate, but not normalize, the time percent of the MSD+EE group when compared to the control group (*P* < 0.05). These results indicated that EE improved MSD-related spatial learning and memory impairment.

### Effect of Enriched Environment and Maternal Sleep Deprivation on BDNF and Syt-1 mRNA Levels

**Figure 4:**
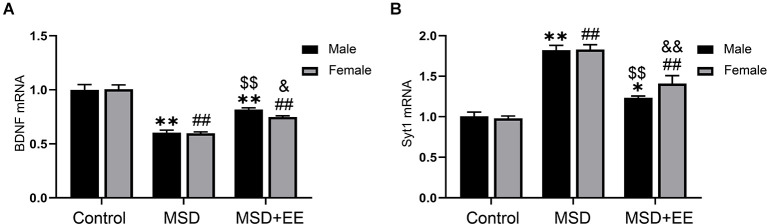
Relative mRNA levels of BDNF and Syt-1 in the hippocampus. **(A)** The mRNA level of BDNF in the hippocampus of the three groups. **(B)** The mRNA level of Syt-1 in the hippocampus of the three groups. *P < 0.05, **P < 0.01 vs. control male; ^##^*P* < 0.01 vs. control female; ^$$^*P* < 0.01 vs. MSD male; ^&^*P* < 0.05, ^&&^*P* < 0.01 vs. MSD female.

A two-way ANOVA showed significant between-group differences in the mRNA levels of BDNF and Syt-1 (BDNF: *F*_(2,42)_ = 99.36, *P* < 0.01; Syt-1: *F*_(2, 42)_ = 102.82, *P* < 0.01; Figures 4A,B). The *post hoc* analysis revealed that the MSD group had a lower BDNF mRNA level and higher Syt-1 mRNA level than the control group (*P*s < 0.01). EE increased the BDNF mRNA level and Syt-1 mRNA level, as shown by the MSD+EE group vs. MSD group comparison (*P*s < 0.05).

### Effect of Enriched Environment and Maternal Sleep Deprivation on BDNF and Syt-1 Protein Levels

**Figure 5:**
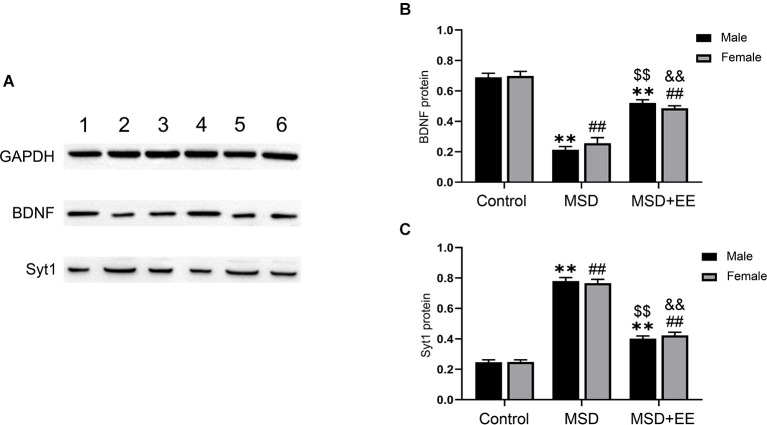
Hippocampal protein levels of BDNF and Syt-1. **(A)** Western blot: band 1: Control-male; band 2: MSD-male; band 3: MSD+EE-male; band 4: Control-female; band 5: MSD-female; band 6: MSD+EE-female. **(B)** The protein level of BDNF in the hippocampus of the three groups. **(C)** The protein level of Syt-1 in the hippocampus of the three groups. **P < 0.01 vs. control male; ^##^*P* < 0.01 vs. control female; ^$$^*P* < 0.01 vs. MSD male; ^&&^*P* < 0.01 vs. MSD female.

We further evaluated the protein levels of BDNF and Syt-1 using Western blotting. A two-way ANOVA showed that the protein levels of BDNF and Syt-1 were significantly different between the three groups (BDNF: *F*_(2,30)_ = 156.01, *P* < 0.01; Syt-1: *F*_(2,30)_ = 379.71, *P* < 0.01; Figures 5A–C). The *post hoc* analysis revealed that MSD decreased the protein level of BDNF and increased the protein level of Syt-1, as revealed by the MSD group vs. control group comparison (*P*s < 0.01). EE increased the BDNF protein level and decreased the Syt-1 protein level in mice from the MSD+EE group relative to the MSD group (*P*s < 0.01).

## Discussion

This study investigated the beneficial effects of EE on MSD-induced anxiety-like behavior and spatial cognition dysfunction, as well as synaptic plasticity markers changes in offspring CD-1 mice. Our results indicate that EE could be a useful strategy to alleviate the anxiety-like behaviors and cognition dysfunction caused by MSD. Preservation of cognitive function was associated with an increase in BDNF and a decrease in Syt-1 in the hippocampus.

Insufficient sleep during pregnancy is a public health problem that brings serious mental and psychological problems to offspring, and also increases the financial burden on society (Oyiengo et al., 2014). Therefore, it is interesting and meaningful to use an MSD rodent model to explore the behavioral phenotypes and their underlying biological mechanisms in MSD-induced offspring. There have been conflicting results on the anxiety-like behavior of MSD-induced offspring. One study suggested that Sprague-Dawley rats born to mothers undergoing sleep deprivation during pregnancy displayed anxiety-like behavior during the EPM and novelty-suppressed feeding task (Peng et al., 2016). Another study reported that Wistar rats exposed to MSD during gestation showed a decrease in anxiety-related behavior during the EPM (Radhakrishnan et al., 2015). In the present study, we found that the offspring of MSD CD-1 mice showed anxiety-like behavior, as indicated by decreased time and entries in the center and open arms during OFT and EPM, separately. It may be that offspring from different strains have different tolerances to MSD. Furthermore, the MWM results suggested that MSD impaired hippocampus-dependent learning and memory, which has been validated in numerous experiments (Zhao et al., 2014; Peng et al., 2016). Moreover, we found that EE reversed the levels of MSD-induced anxiety, and alleviated—yet did not normalize—MSD-induced cognitive dysfunction. The hippocampus is an important brain region for learning and memory, and its morphology and synaptic function are more susceptible to stress and more difficult to repair than emotion-related regions (Guan et al., 2004; Ruskin et al., 2004). To the best of our knowledge, our experiment is the first to demonstrate the beneficial effect of EE on MSD-associated anxiety-like behavior and cognitive impairment.

BDNF is widely expressed in the hippocampus and has potent effects on cognitive function (Autry and Monteggia, 2012). *In vitro* study, BDNF can promote the survival, proliferation, and differentiation of neural stem cells (Chen et al., 2013; Hachem et al., 2015). *In vivo* study, the heterozygous (*BDNF+/–*) mice with knockout of *BDNF* showed a decrease in cell proliferation and survival in the dentate gyrus of the hippocampus (Lee et al., 2002). The (*BDNF^2lox^/BDNF^2lox^/CaMKII-cre*) mice with conditional knockout of *BDNF* in mature neurons exhibited an impaired dendritic development without affecting cell proliferation and differentiation (Choi et al., 2009). The inconsistent results regarding the effects of BDNF are due to differences in experimental methods and gene knockout techniques. A previous study had demonstrated that the C57BL/6 mice exposed to isoflurane during aging exhibited cognitive deficits, which were accompanied by inhibition of the BDNF pathway and downregulation of synaptic plasticity markers in the hippocampus (Wu et al., 2016). Similarly, our results showed that MSD resulted in impaired cognitive function accompanied by a downregulation of BDNF. Furthermore, a previous study demonstrated that adolescent enriched environment could alleviate sleep deprivation-associated cognition dysfunction by restoring BDNF expression levels in male Wistar rats (Ghaheri et al., 2022). Our Western blotting and real-time fluorescence quantitative polymerase chain reaction results have suggested that EE increases BDNF mRNA and protein levels in the hippocampus of MSD mice. A previous study showed that long-term treatment of hippocampal slice cultures with BDNF increased the number of docked vesicles at hippocampal CA1 synapses and increased the protein levels of synaptotagmin, synaptophysin, and synaptobrevin (Tartaglia et al., 2001). The interaction between the synaptic vesicle-associated proteins and BDNF might trigger the imbalance of synaptic plasticity that occurs in cognitive impairment. The downregulation of Syt-1 has been implicated in chronic brain hypoperfusion-associated presynaptic plasticity dysfunction and treadmill exercise training improved hippocampus-associated learning and memory by upregulation of Syt-1, which indicated that the expression level of Syt-1 was positive with cognitive function (Liu et al., 2009; Yan et al., 2020). Surprisingly, our results showed that MSD-induced cognitive impairment was associated with increased Syt-1 expression. The results are in accordance with a previous report that upregulation of Syt-1 in the hippocampus has been found to cause neuron damage associated with prenatal stress- and hypothyroidism-induced cognitive impairment (Vara et al., 2002; Jia et al., 2010). Moreover, EE could improve MSD associated-cognitive deficits by reducing Syt-1 expression, which was consistent with our previous study that exposure to an EE from adolescence improved age-associated cognitive decline by downregulating the expression of Syt-1 (Zhang et al., 2022). The contradictory results regarding the relationship between Syt-1 expression level and cognitive function could be a result of differences in the specific mechanisms leading to cognitive impairment in different pathological models.

Importantly, the mechanisms underlying MSD-related cognitive impairment involve not only changes in synaptic markers but also alterations in inflammation and the hypothalamic-pituitary-adrenal axis (Zhao et al., 2014; Ehichioya et al., 2022). Previous studies suggested that EE could improve cognitive dysfunction by altering pro-inflammatory cytokines and hyperactivity of the hypothalamic-pituitary-adrenal axis (Delanogare et al., 2020; Keymoradzadeh et al., 2020). The effects of EE on inflammatory and hypothalamic-pituitary-adrenal markers should be further explored in MSD models. Moreover, we found that EE attenuated MSD-associated impaired cognition, but did not fully reverse it. Further studies could examine the effect of drugs and other non-drug treatments, such as exercise, on MWM performance in MSD-induced mice (Liu et al., 2009).

Our study has limitations. First, we only used Western blotting and real-time fluorescence quantitative polymerase chain reaction to evaluate the expression levels of BDNF and Syt-1, and did not use immunohistochemistry to quantitatively analyze the effect of EE on the expression levels of BDNF and Syt-1 in different subregions of the hippocampus. Second, we did not further assess hippocampal synaptic plasticity resulting from changes in synaptic plasticity markers by patch-clamp technique. Third, we did not use RNA interference technology to reduce BDNF and increase Syt-1 to verify the targets of EE.

## Conclusion

Our study demonstrated that MSD-induced offspring exhibited anxiety-like behavior, cognitive impairment, and BDNF and Syt-1 expression. Notably, sleep deprivation during pregnancy induced randomly without analyzing the circadian rhythm and their activity pattern. Access to EE alleviated anxiety-like behavior and cognitive impairment in offspring from the MSD group. The improved cognitive function can be partially explained by an increase in BDNF and a decrease in Syt-1 in the hippocampus. Thus, EE treatment may have utility for the prevention of the development of anxiety-like behaviors and recovery from cognitive deficits following sleep deprivation during pregnancy.

## Data Availability Statement

The data used to support the findings of this study are available from the corresponding author upon request.

## Ethics Statement

The animal study was reviewed and approved and all animal experiments were carried out in compliance with the guidelines for humane treatment set by the Association of Laboratory Animal Sciences and the Center for Laboratory Animal Sciences at Anhui Medical University (No. LLSC20190710).

## Author Contributions

Y-MZ and Y-ZC designed the study, performed behavioral tests, and drafted the manuscript. Y-TW and R-MW were responsible for western blotting and real-time fluorescence quantitative polymerase chain reaction. Y-JG and X-YK analyzed the data and made graph. X-YL revised the manuscript and were responsible for the completeness and accuracy of the data. All authors contributed to the article and approved the submitted version.

## Conflict of Interest

The authors declare that the research was conducted in the absence of any commercial or financial relationships that could be construed as a potential conflict of interest.

## Publisher’s Note

All claims expressed in this article are solely those of the authors and do not necessarily represent those of their affiliated organizations, or those of the publisher, the editors and the reviewers. Any product that may be evaluated in this article, or claim that may be made by its manufacturer, is not guaranteed or endorsed by the publisher.

## References

[B1] Alwis D. S. Rajan R. (2014). Environmental enrichment and the sensory brain: the role of enrichment in remediating brain injury. Front. Syst. Neurosci. 8:156. 10.3389/fnsys.2014.00156 25228861 PMC4151031

[B2] Autry A. E. Monteggia L. M. (2012). Brain-derived neurotrophic factor and neuropsychiatric disorders. Pharmacol. Rev. 64, 238–258. 10.1124/pr.111.005108 22407616 PMC3310485

[B3] Chanaday N. L. Nosyreva E. Shin O. H. Zhang H. Aklan I. Atasoy D.. (2021). Presynaptic store-operated Ca^2+^ entry drives excitatory spontaneous neurotransmission and augments endoplasmic reticulum stress. Neuron 109, 1314–1332.e5. 10.1016/j.neuron.2021.02.023 33711258 PMC8068669

[B4] Chang J. J. Pien G. W. Duntley S. P. Macones G. A. (2010). Sleep deprivation during pregnancy and maternal and fetal outcomes: is there a relationship? Sleep Med. Rev. 14, 107–114. 10.1016/j.smrv.2009.05.001 19625199 PMC2824023

[B5] Chen B. Y. Wang X. Wang Z. Y. Wang Y. Z. Chen L. W. & Luo Z. J. (2013). Brain-derived neurotrophic factor stimulates proliferation and differentiation of neural stem cells, possibly by triggering the Wnt/β-catenin signaling pathway. J. Neurosci. Res. 91, 30–41. 10.1002/jnr.23138 23023811

[B6] Choi S. H. Li Y. Parada L. F. & Sisodia S. S. (2009). Regulation of hippocampal progenitor cell survival, proliferation and dendritic development by BDNF. Mol. Neurodegener. 4:52. 10.1186/1750-1326-4-52 20025751 PMC2806355

[B7] Delanogare E. de Souza R. M. Rosa G. K. Guanabara F. G. Rafacho A. Moreira E. (2020). Enriched environment ameliorates dexamethasone effects on emotional reactivity and metabolic parameters in mice. Stress 23, 466–473. 10.1080/10253890.2020.1735344 32107952

[B8] Ehichioya D. E. Tahajjul Taufique S. K. Anigbogu C. N. Jaja S. I. (2022). Effect of rapid eye movement sleep deprivation during pregnancy on glucocorticoid receptor regulation of HPA axis function in female offspring. Brain Res. 1781:147823. 10.1016/j.brainres.2022.147823 35151654

[B9] Entringer S. Buss C. Wadhwa P. D. (2012). Prenatal stress, telomere biology and fetal programming of health and disease risk. Sci. Signal. 5:pt12. 10.1126/scisignal.2003580 23112344

[B10] Ghaheri S. Panahpour H. Abdollahzadeh M. & Saadati H. (2022). Adolescent enriched environment exposure alleviates cognitive impairments in sleep-deprived male rats: role of hippocampal brain-derived neurotrophic factor. Int. J. Dev. Neurosci. 82, 133–145. 10.1002/jdn.10165 34937120

[B11] Guan Z. Peng X. Fang J. (2004). Sleep deprivation impairs spatial memory and decreases extracellular signal-regulated kinase phosphorylation in the hippocampus. Brain Res. 1018, 38–47. 10.1016/j.brainres.2004.05.032 15262203

[B12] Hachem L. D. Mothe A. J. Tator C. H. (2015). Effect of BDNF and other potential survival factors in models of *in vitro* oxidative stress on adult spinal cord-derived neural stem/progenitor cells. Biores. Open Access 4, 146–159. 10.1089/biores.2014.0058 26309791 PMC4497651

[B13] Izci-Balserak B. Keenan B. T. Corbitt C. Staley B. Perlis M. Pien G. W. (2018). Changes in sleep characteristics and breathing parameters during sleep in early and late pregnancy. J. Clin. Sleep Med. 14, 1161–1168. 10.5664/jcsm.7216 29991418 PMC6040782

[B14] Jia N. Yang K. Sun Q. Cai Q. Li H. Cheng D.. (2010). Prenatal stress causes dendritic atrophy of pyramidal neurons in hippocampal CA3 region by glutamate in offspring rats. Dev. Neurobiol. 70, 114–125. 10.1002/dneu.20766 19950194

[B15] Keymoradzadeh A. Hedayati Ch M. Abedinzade M. Gazor R. Rostampour M. Taleghani B. K. (2020). Enriched environment effect on lipopolysaccharide-induced spatial learning, memory impairment and hippocampal inflammatory cytokine levels in male rats. Behav. Brain Res. 394:112814. 10.1016/j.bbr.2020.112814 32707137

[B16] Leal G. Afonso P. M. Salazar I. L. Duarte C. B. (2015). Regulation of hippocampal synaptic plasticity by BDNF. Brain Res. 1621, 82–101. 10.1016/j.brainres.2014.10.019 25451089

[B17] Lee J. Duan W. Mattson M. P. (2002). Evidence that brain-derived neurotrophic factor is required for basal neurogenesis and mediates, in part, the enhancement of neurogenesis by dietary restriction in the hippocampus of adult mice. J. Neurochem. 82, 1367–1375. 10.1046/j.1471-4159.2002.01085.x 12354284

[B18] Liu Y. F. Chen H. I. Wu C. L. Kuo Y. M. Yu L. Huang A. M.. (2009). Differential effects of treadmill running and wheel running on spatial or aversive learning and memory: roles of amygdalar brain-derived neurotrophic factor and synaptotagmin I. J. Physiol. 587, 3221–3231. 10.1113/jphysiol.2009.173088 19451201 PMC2727033

[B19] Micheli K. Komninos I. Bagkeris E. Roumeliotaki T. Koutis A. Kogevinas M.. (2011). Sleep patterns in late pregnancy and risk of preterm birth and fetal growth restriction. Epidemiology 22, 738–744. 10.1097/EDE.0b013e31822546fd 21734587

[B20] Murueta-Goyena A. Morera-Herreras T. Miguelez C. Gutiérrez-Ceballos A. Ugedo L. Lafuente J. V.. (2019). Effects of adult enriched environment on cognition, hippocampal-prefrontal plasticity and NMDAR subunit expression in MK-801-induced schizophrenia model. Eur. Neuropsychopharmacol. 29, 590–600. 10.1016/j.euroneuro.2019.03.009 30926324

[B21] Oyiengo D. Louis M. Hott B. Bourjeily G. (2014). Sleep disorders in pregnancy. Clin. Chest Med. 35, 571–587. 10.1016/j.ccm.2014.06.012 25156772

[B22] Peng Y. Wang W. Tan T. He W. Dong Z. Wang Y. T.. (2016). Maternal sleep deprivation at different stages of pregnancy impairs the emotional and cognitive functions and suppresses hippocampal long-term potentiation in the offspring rats. Mol. Brain 9:17. 10.1186/s13041-016-0197-3 26876533 PMC4753670

[B23] Radhakrishnan A. Aswathy B. S. Kumar V. M. Gulia K. K. (2015). Sleep deprivation during late pregnancy produces hyperactivity and increased risk-taking behavior in offspring. Brain Res. 1596, 88–98. 10.1016/j.brainres.2014.11.021 25446439

[B24] Ruskin D. N. Liu C. Dunn K. E. Bazan N. G. LaHoste G. J. (2004). Sleep deprivation impairs hippocampus-mediated contextual learning but not amygdala-mediated cued learning in rats. Eur. J. Neurosci. 19, 3121–3124. 10.1111/j.0953-816X.2004.03426.x 15182321

[B25] Sedov I. D. Cameron E. E. Madigan S. Tomfohr-Madsen L. M. (2018). Sleep quality during pregnancy: a meta-analysis. Sleep Med. Rev. 38, 168–176. 10.1016/j.smrv.2017.06.005 28866020

[B26] Spriggs M. J. Thompson C. S. Moreau D. McNair N. A. Wu C. C. Lamb Y. N.. (2019). Human sensory LTP predicts memory performance and is modulated by the BDNF Val66Met polymorphism. Front. Hum. Neurosci. 13:22. 10.3389/fnhum.2019.00022 30828292 PMC6384276

[B27] Tartaglia N. Du J. Tyler W. J. Neale E. Pozzo-Miller L. Lu B. (2001). Protein synthesis-dependent and -independent regulation of hippocampal synapses by brain-derived neurotrophic factor. J. Biol. Chem. 276, 37585–37593. 10.1074/jbc.M101683200 11483592

[B28] Thome J. Pesold B. Baader M. Hu M. Gewirtz J. C. Duman R. S.. (2001). Stress differentially regulates synaptophysin and synaptotagmin expression in hippocampus. Biol. Psychiatry 50, 809–812. 10.1016/s0006-3223(01)01229-x 11720700

[B29] Vara H. Martínez B. Santos A. & Colino A. (2002). Thyroid hormone regulates neurotransmitter release in neonatal rat hippocampus. Neuroscience 110, 19–28. 10.1016/s0306-4522(01)00541-3 11882369

[B30] Wang H. Xu X. Xu X. Gao J. Zhang T. (2020). Enriched environment and social isolation affect cognition ability via altering excitatory and inhibitory synaptic density in mice hippocampus. Neurochem. Res. 45, 2417–2432. 10.1007/s11064-020-03102-2 32748366

[B31] Wilson D. L. Barnes M. Ellett L. Permezel M. Jackson M. Crowe S. F. (2011). Decreased sleep efficiency, increased wake after sleep onset and increased cortical arousals in late pregnancy. Aust. N Z J. Obstet. Gynaecol. 51, 38–46. 10.1111/j.1479-828X.2010.01252.x 21299507

[B33] Wu Y. F. Zhang Y. M. Ge H. H. Ren C. Y. Zhang Z. Z. Cao L.. (2020). Effects of embryonic inflammation and adolescent psychosocial environment on cognition and hippocampal staufen in middle-aged mice. Front. Aging Neurosci. 12:578719. 10.3389/fnagi.2020.578719 33024434 PMC7516039

[B32] Wu J. Zhang M. Li H. Sun X. Hao S. Ji M.. (2016). BDNF pathway is involved in the protective effects of SS-31 on isoflurane-induced cognitive deficits in aging mice. Behav. Brain Res. 305, 115–121. 10.1016/j.bbr.2016.02.036 26944333

[B34] Xie L. Kang H. Xu Q. Chen M. J. Liao Y. Thiyagarajan M.. (2013). Sleep drives metabolite clearance from the adult brain. Science 342, 373–377. 10.1126/science.1241224 24136970 PMC3880190

[B35] Yan M. L. Zhang S. Zhao H. M. Xia S. N. Jin Z. Xu Y.. (2020). MicroRNA-153 impairs presynaptic plasticity by blocking vesicle release following chronic brain hypoperfusion. Cell Commun. Signal. 18:57. 10.1186/s12964-020-00551-8 32252776 PMC7137307

[B36] Yang W. Zhou X. Zimmermann H. R. Ma T. (2021). Brain-specific suppression of AMPKα2 isoform impairs cognition and hippocampal LTP by PERK-mediated eIF2α phosphorylation. Mol. Psychiatry 26, 1880–1897. 10.1038/s41380-020-0739-z 32366952 PMC8054310

[B37] Yu Z. Wang J. Zhang P. Wang J. Cui J. Wang H. (2020). Enriched environment improves sevoflurane-induced cognitive impairment during late-pregnancy via hippocampal histone acetylation. Braz. J. Med. Biol. Res. 53:e9861. 10.1590/1414-431x20209861 32813852 PMC7433840

[B38] Zhang X. Shi X. Wang J. Xu Z. He J. (2021). Enriched environment remedies cognitive dysfunctions and synaptic plasticity through NMDAR-Ca^2+^-Activin A circuit in chronic cerebral hypoperfusion rats. Aging (Albany NY) 13, 20748–20761. 10.18632/aging.203462 34462377 PMC8436900

[B39] Zhang Z. Z. Zeng L. P. Chen J. Wu Y. F. Wang Y. T. Xia L.. (2022). Long-term environmental enrichment relieves dysfunctional cognition and synaptic protein levels induced by prenatal inflammation in older CD-1 mice. Neural Plast. 2022:1483101. 10.1155/2022/1483101 35574247 PMC9106518

[B40] Zhao Q. Peng C. Wu X. Chen Y. Wang C. You Z. (2014). Maternal sleep deprivation inhibits hippocampal neurogenesis associated with inflammatory response in young offspring rats. Neurobiol. Dis. 68, 57–65. 10.1016/j.nbd.2014.04.008 24769004

[B41] Zhuang Z. Q. Zhang Z. Z. Zhang Y. M. Ge H. H. Sun S. Y. Zhang P.. (2021). A long-term enriched environment ameliorates the accelerated age-related memory impairment induced by gestational administration of lipopolysaccharide: role of plastic mitochondrial quality control. Front. Cell. Neurosci. 14:559182. 10.3389/fncel.2020.559182 33613195 PMC7886998

